# Potential Role of Cyr61 Induced Degeneration of Human Müller Cells in Diabetic Retinopathy

**DOI:** 10.1371/journal.pone.0109418

**Published:** 2014-10-16

**Authors:** Fen Zhou, Yikui Zhang, Ding Chen, Zhitao Su, Ling Jin, Lei Wang, Zhixiang Hu, Zhisheng Ke, Zongming Song

**Affiliations:** 1 Eye Hospital, Wenzhou Medical University, Wenzhou, Zhejiang, China; 2 Eye Center, Second Affiliated Hospital, College of Medicine, Zhejiang University, China; Medical University of South Carolina, United States of America

## Abstract

The degeneration of Müller cells has been recognized to involve in the pathogenesis of diabetic retinopathy. However, the mechanism is not yet clear. This study is to explore the potential role of Cyr61, a secreted signaling protein in extracellular matrix, in inducing human Müller cell degeneration in diabetic retinopathy (DR). Twenty patients with proliferative diabetic retinopathy (PDR) and twelve non-diabetic patients were recruited for this study. Vitreous fluid was collected during vitrectomy surgery for Cyr61 ELISA. Human Müller cell line MIO-M1 were cultured to be subconfluent, and then treated with glucose (0–20 mM) or Cyr61 (0–300 ng/ml). Cyr61 expression induced by increasing concentrations of glucose was evaluated by RT-qPCR and Western blot. Effects of Cyr61 on Müller cells viability, migration and apoptosis were observed by MTT assay, Transwell assay, and TUNEL assay. Vitreous Cyr61 levels were observed to be 8-fold higher in patients with PDR (3576.92±1574.58 pg/mL), compared with non-diabetic controls (436.14±130.69 pg/mL). Interestingly, the active PDR group was significantly higher than the quiescent PDR group (P<0.01). In retinal Müller cells culture, high glucose significantly and dose-dependently elevated Cyr61 expression at both mRNA and protein levels. Cyr61 at high concentrations dose-dependently inhibited the viability and migration of Müller cells. TUNEL assay further revealed that high concentration of Cyr61 significantly promoted the cell apoptosis. In conclusion, these findings demonstrated for the first time that the expression of Cyr61 was elevated by high glucose in Müller cells, and Cyr61 inhibited cell viability and migration while induced apoptosis, suggesting the potential role of Cyr61 in Müller cell degeneration. The elevated Cyr61 levels in vitreous fluid of PDR patients further support its role in diabetic retinopathy (DR).

## Introduction

Diabetic retinopathy (DR) is one of the leading causes of visual impairment [1], [2]. Its pathophysiology involves the degeneration of the retinal neurons and dysfunction of the retinal microvasculature, followed by macular edema and neovascularization of the retina. Many studies mainly focus on the angiogenic factors, such as vascular endothelial growth factor (VEGF). However, less attention has been paid to the degeneration of the Müller cells, which anatomically and physiologically account for additional pathologic mechanisms in diabetic retinopathy [3]–[5].

Müller cells are the primary retinal glial cells, radically spanning within the full thickness of the retina. Processes of Müller cells rise from the outer and inner trunks, enveloping neurons, synapses and blood vessels, and contributing to the organization of the retina and the formation of blood-retinal barrier. Damage of the blood-retinal barrier is thought to be involved in the pathology of diabetic retinopathy [6], [7]. Müller cells also express ion channels, water channels and glutamate transporters that strictly regulate the retinal microenvironment, dysfunction of which leads to macular edema and the toxicity of the retinal neurons [8]. In addition, Müller cells could secret several trophic factors, such as glial cell line-derived neurotrophic factor and basic fibroblast growth factor, to protect the photoreceptors [9]–[11]. Thus the degeneration of Müller cells may accelerate the apoptosis of the neurons in pathologic stimulus.

It has been recognized that the degeneration or apoptosis of Müller cells plays an important part in the process of DR [12]. However, the exact mechanism of how Müller cells degenerates is still unknown. Previous studies found that the accumulation of Cyr61 could cause the senescence and apoptosis of the fibroblast in wound healing [13], into which the Müller cells might transdifferenciate under pathologic stimulus like hyperglycemia. Also, the Cyr61 in vitreous fluid of patients with DR is higher than patients without DR. Thus we hypothesize that Cyr61 might account partially for the degeneration of the Müller cells.

Cyr61 is a 40 kD secreted cysteine-rich (Cyr) heparin-binding protein, which could regulate cell proliferation, migration, adhesion, and apoptosis [14], [15]. Cyr61 belongs to CCN family consisting of Cyr61 (CCN1), CTGF (CCN2), NOV (CCN3), WISP-1 (CCN4), WISP-2 (CCN5), and WISP-3 (CCN6) [16]–[18]. The importance of Cyr61 in the retina has been increasingly noticed for its role in retinal angiopathy, since Cyr61 has potent angiogenic activity on endothelia cells and ability to induce neovascularization [19]–[21]. It has been suggested that Cyr61 may act in a synergetic manner in angiogenesis with VEGF [20].

Cyr61 is observed to be highly expressed in the retina of diabetic animal model [19]–[21] and vitreous fluid of patients with DR [20]. Previous study thought that Cyr61 was largely secreted by retinal endothelium cells and pericyte [22]. Cyr61 has been reported to mainly exist in the outer plexiform layer, inner plexiform layer, ganglion cells layer and photoreceptor layer, while its expression becomes higher in inner plexiform layer, ganglion cells layer in diabetic animal model [19]. According to the wide structural range of the Müller cells, we further hypothesize that the elevated expression of Cyr61 in DR is partially from Müller cells.

In all, both the degeneration of Müller cell and high expression of Cyr61 take critical parts in the pathogenesis of DR. This study was to seek answers to these following questions: Can Müller cells express Cyr61? How does the expression of Cyr61 in Müller cells be affected in high glucose condition? What are the effects of various concentrations of Cyr61 on the viability of Müller cells?

## Materials and Methods

### Patients

This study was performed in accordance with the Helsinki Declaration. The ethics committee of Wenzhou Medical University approved the protocol for collection and testing of vitreous fluid. Thirty two patients who received vitrectomy at the Eye Hospital of Wenzhou Medical University from December 1, 2012 to March 30, 2013 were enrolled in this study after signing a consent form. According to the grouping method in a previously published paper [23], 20 diabetic patients with PDR were divided into active PDR group (10 patients) and quiescent PDR group (10 patients). Twelve control subjects, including 5 with idiopathic epiretinal membranes and 7 with idiopathic macular holes were enrolled. Vitreous fluid samples (0.5 to 1 mL) were harvested at the start of vitrectomy. The vitreous samples were transferred to a sterile tube and placed immediately on ice, and then centrifuged at 15,000 rpm for 15 minutes at 4°C to discard cells and debris. Supernatants were frozen at −80°C before use.

### Diagnosis criteria

(1) Diagnostic criteria for PDR: ophthalmoscopy to determine the presence of one or more of the following signs in the retina of DR patients: neovascularization, pre-retinal hemorrhages, vitreous hemorrhages, fibrovascular tissue proliferation, tractional retinal detachments. According to classification by Aiello [23] and You [24], we further defined active PDR as PDR with perfused multi-branching iris or preretinal capillaries, while quiescent PDR as PDR with fully regressed proliferation or with only nonperfused, gliotic vessels. (2) Diagnostic criteria for idiopathic epiretinal membranes: ocular coherence tomography (OCT) to elucidate the presence of an ERM, in patients without any associated diseases or known history. (3) Diagnostic criteria for idiopathic macular holes: fundus image or OCT to visualize a full-thickness macular hole which is characterized by a well-defined round or oval lesion in the macula, in patients with no history of ocular trauma.

### Reagents

Dulbeccos Modified Eagle Medium (DMEM), 10% fetal bovine serum (FBS), penicillin and streptomycin were purchased from Gibco (CA, USA); D-glucose was purchased from Sigma (MO, USA); Cyr61 recombinant protein was purchased from Peprotech Inc. (Rocky Hill, NJ, USA). Cyr61 polyclonal antibody was purchased from Abcam (Cambridge, UK).

### Müller cell culture and treatment with glucose or Cyr61

Human Müller cell line MIO-M1 (Moorfields/Institute of Ophthalmology- Müller 1) was obtained from American Type Culture Collection (ATCC). The cells were cultured in DMEM with 10% fetal bovine serum, 2 mmol/L glutamine, 100 ug/ml penicillin, and 100 ug/ml streptomycin. In order to measure the effect of high glucose on the expression of Cyr61 in Müller cells, these cells were treated with different concentrations of glucose (5–20 mM) for 0, 4, 6, 8, 24 h to determine Cyr61 expression at mRNA and protein levels. For other study investigating the effect of Cyr61 on Müller cell degeneration, these cells, cultured in the same medium without additional glucose, were incubated with different concentrations (0–300 ng/ml) of Cyr61 to evaluate cell viability, migration, apoptosis by MTT assay, Transwell assay and TUNEL assay, respectively.

### Cell viability assay

Cell proliferation was examined using MTT (3-[4, 5-Dimethylthiazol-2-yl]-2, 5-diphenyltetrazolium bromide; Thiazolyl blue) assay. Müller cells were seeded in 96-well plates (5×10^3^ cells per well), and were serum-starved for 4 hours. Then Cyr61 in different concentrations (0–300 ng/ml) was added with different incubation periods (24 h, 48 h,). 20 µl MTT (5 mg/ml) was added to each well, and the cells were cultured for another 4 hours. Supernatant was discarded, 150 µl DSMO was added and the plate was shaken for 10 minutes. The reaction product amount was determined by measuring absorbance at 490 nm with a spectrophotometric reader M5 (Molecular Devices, USA)

### Cell migration assay

Cell migration was determined by Transwell assay. The effect of Cyr61 on cell migration was assessed using 24-well Transwell chambers (Costar, American), and the pore size was 8.0 mm. After starving in 0.5% FBS medium for 4 hours, Müller cells were seeded in the upper chamber (8×10^3^ cells per well), and 500 µl culture medium with 10% FBS was plated in the lower chamber. Different concentrations of Cyr61 (0–300 ng/ml) was added in the lower chamber. The membrane was removed after 12 hours of incubation, and was fixed with 4% paraformaldehyde for 20 min and stained with crystal violet, washed three times with phosphate buffer saline (PBS). The remaining cells on the upper surface of the filter were removed by wiping with a cotton swab. Cells migration was quantified by the number of cells that migrated across the filter toward the lower surface in five random fields per filter under microscope.

### Cell apoptosis assay

Müller cell apoptosis was detected by the TUNEL (terminal deoxynucleotidyl transferase dUTP nick-end labeling). In situ detection of DNA fragments by terminal deoxyribonucleotide transferase (TdT)-mediated dUTP nick end labeling (TUNEL) was performed using the one-step TUNEL apoptosis assay kit (Beyotime, China). According to the manufacturer's instructions, Müller cells were also stained with 4′, 6′-diamino-2-phenylin-dole (DAPI, blue fluorescence; Molecular Probes) to visualize the cell nuclear. The apoptosis rate was determined by TUNEL percentage, which was calculated by the number of TUNEL-positive cells, and the average was calculated.

### Detection of Cyr61 in vitreous fluid of patients with PDR

The expression level of Cyr61 in vitreous body was measured by a human Cyr61 enzyme-linked immunosorbent assays (ELISA) kit (Human Cyr61 ELISA Kit, Catalog No.CSB-E13884h, cusabio, China). Prepare all reagents, working standards and samples. Add 100 µl of Standard, Blank, or Sample per well. Incubate for 2 hours at 37°C. Cover with the adhesive strip provided. Remove the liquid of each well, don't wash. Add 100 µl of Biotin-antibody to each well. Cover with a new adhesive strip. Incubate for 1 hour at 37°C. Aspirate each well and wash, repeating the process three times for a total of three washes. Add 100 µl of HRP-avidin to each well. Incubate for 1 h at 37°C. Repeat the aspiration and wash five times. Add 90 µl of TMB Substrate to each well and the plate was developed in darkness at room temperature for 15–30 minutes. Add 50 µl of Stop Solution to each well and concentrations were determined at 450 nm (correction 550 nm) using spectrophotometric reader M5 (Molecular Devices, USA) within 5 minutes. Cyr61 level was expressed as pg/mL.

### RNA extraction, reverse transcription, and real time-quantitative (RT-qPCR)

Total RNA was extracted with a RNeasy Mini Kit (Qiagen, VA, USA) according to the manufacturer's instructions, quantified with a NanoDrop ND-1000 spectrophotometer (Thermo Scientific), and stored at −80°C before use. The cDNA was synthesized from 1 µg of total RNA using random primer and M-MLV reverse transcriptase. RT-qPCR was performed with 15 µl reaction volume which contained 50 ng of cDNA, 20×TaqMan Gene Expression Master Mix, TaqMan Gene Expression Assay. TaqMan Gene Expression Assay were used: GAPDH (HS02758991_g1), Cyr61 (HS00998500_g1). RT-qPCR used the Applied Biosystems 7500 Fast Real-Time PCR System (Applied Biosystems, CA, USA). The results were analyzed by the comparative threshold cycle (Ct) method and normalized by GAPDH as an internal control.

### Western blotting

Proteins were extracted from cell lysates. For Western blot analysis, the cell lysates (50 mg per lane, measured by a BCA protein assay kit) were mixed with 1×SDS reducing sample buffer and boiled for 5 min before loading. For the purpose of normalization an equivalent amount (20 µl) of soluble protein from cell lysates was loaded onto the gel. The proteins were separated on an SDS polyacrylamide gel and electronically transferred to NC membranes. The membranes were blocked with 5% nonfat milk in PBST (PBS and 1%Tween-20) for 2 h at room temperature and incubated, first with primary antibodies against Cyr61 (1∶1000, 2 mg/ml) or GAPDH (1∶5000, 1 mg/ml) overnight at 4°C, and then with goat anti-rabbit secondary antibody for 2 h at room temperature.

### Statistical analysis

Statistical analysis was performed using SPSS 19.0 statistical software. Data were expressed as the mean ± SD. Differences between the means of experimental and respective control groups were calculated by Mann-Whitney U test. Human Müller cells proliferation, migration in different groups were analyzed using one-way ANOVA. Correlation analysis among cell viability, apoptosis and migration was performed by Pearson Correlation. A P-value <0.05 was considered to be statistically significant.

## Results

### Vitreous fluid Cyr61 levels in PDR and non-diabetic patients

Vitreous fluid samples were harvested from 20 patients with PDR and 12 patients with non-diabetic ocular diseases as controls. In the PDR group, there were nine male and eleven female, aging from 48 to 73 (57.26±9.16) years old. In the non-diabetic control group, there were five male and seven female, ages ranged from 48 to 76 (65.17±8.28) years old. There was no statistically significant difference in age between the non-diabetic control group and the diabetic patients, with the P = 0.136 by Mann-Whitney U test.

**Figure 1:**
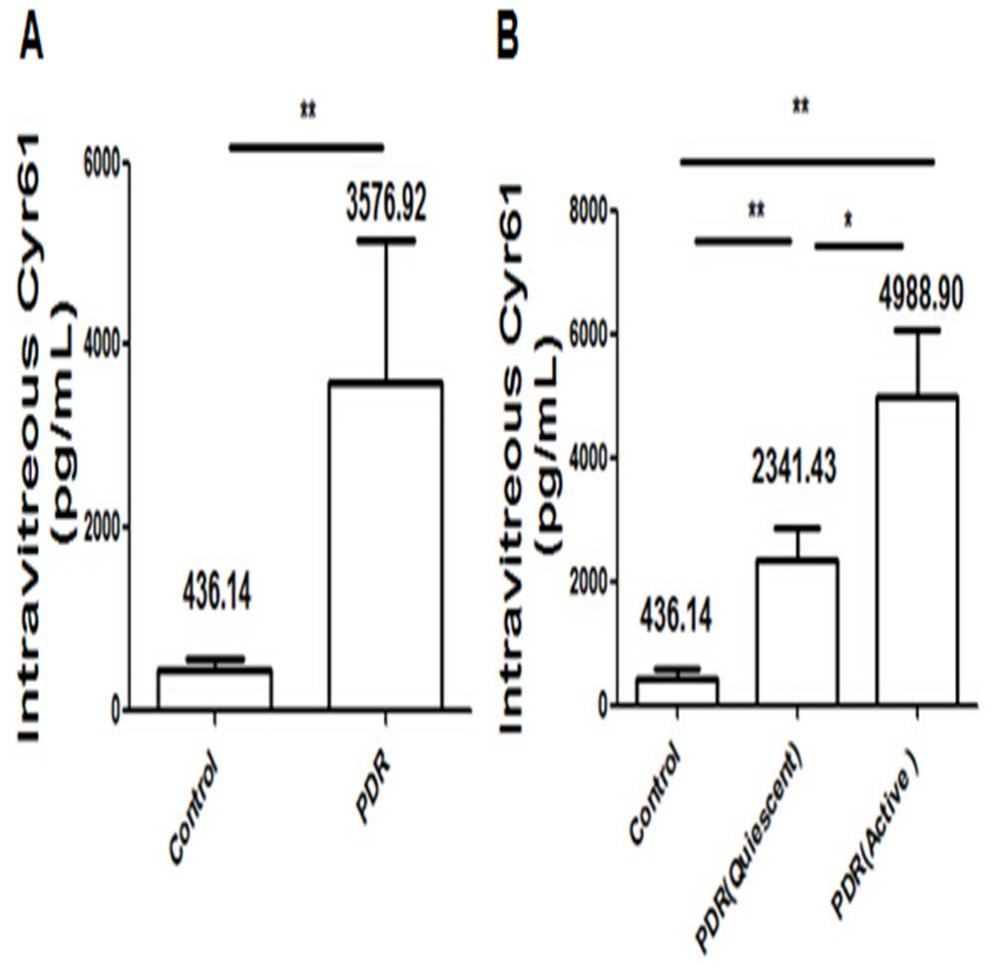
Vitreous levels of Cyr61 in PDR and non-diabetic patients were determined by ELISA. A) Vitreous concentration of Cyr61 in patients with PDR was higher than with non-diabetic disease. The difference was significant. B) Vitreous concentration of Cyr61 in patients with active PDR was significantly higher than with quiescent PDR. Vitreous concentration of Cyr61 in patients with quiescent PDR was higher than with non-diabetic disease. The difference was significant. Results shown are the mean ± SD of three independent experiments. * P<0.05; ** P<0.01.

Vitreous concentration of Cyr61 measured by ELISA in patients with PDR was 3576.92±1574.58 pg/mL, largely higher than that of non-diabetic patients (436.14±130.69 pg/mL). The difference was significant (Mann–Whitney U test, *P* = 0.002). We further observed that vitreous Cyr61 levels in patients with active PDR (4988.90±1058.80 pg/mL) were higher than that in patients with quiescent PDR (2341.43±509.44 pg/mL). The difference was significant (Mann–Whitney U test, *P* = 0.013) (Figure 1).

### mRNA expression and protein production of Cyr61 in Müller cells exposed to glucose

**Figure 2:**
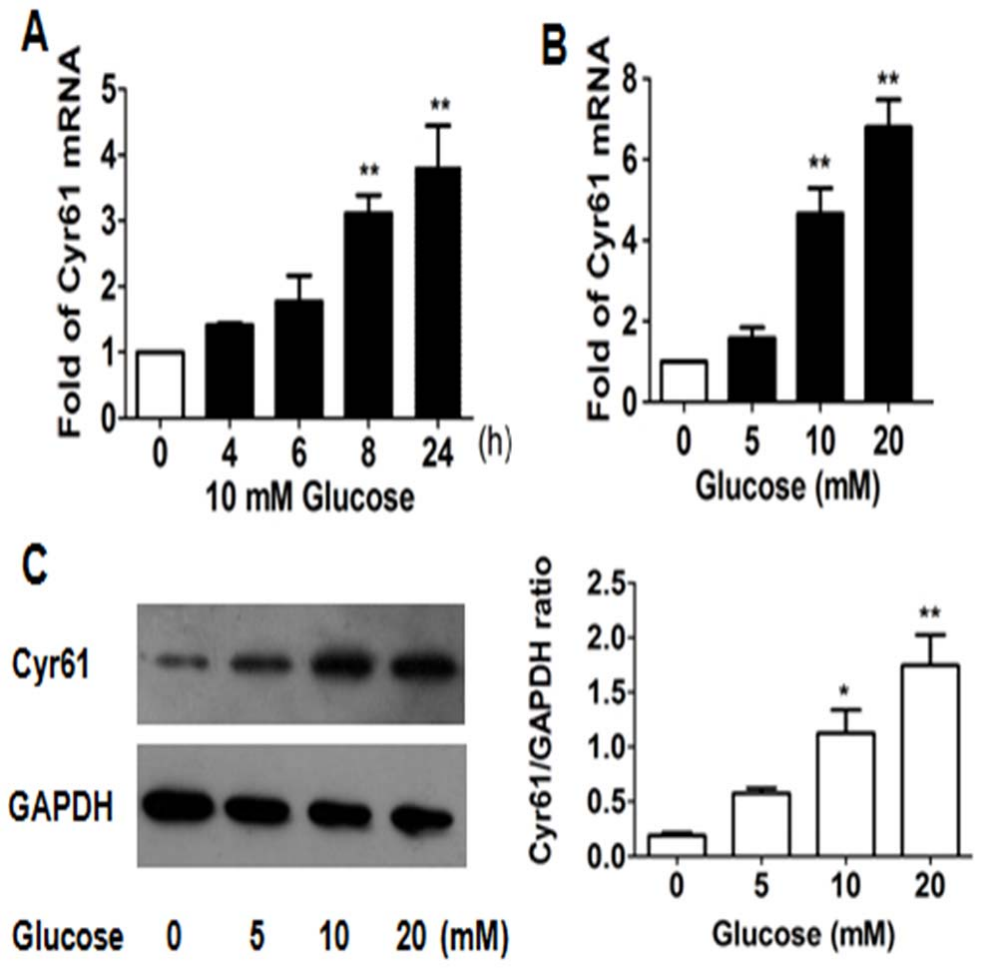
Cyr61 expression in Müller cells was observed at mRNA and protein level. A) Cells were harvested for total mRNA. Cyr61 mRNA levels was assessed by RT-qPCR at 10 mM glucose for 0–24 h. There was a time-dependent increase in expression of Cyr mRNA. B) To further understand the interaction of Cyr61 and high glucose, we examined the Cyr61 expression in Müller cells exposed to different glucose concentrations (0–20 mM) for 8 h at mRNA level. There was a does-dependent increase in expression of Cyr mRNA C) Cyr61 protein expression of Müller cells under different glucose concentrations (0–20 mM) was assessed by Western blot analysis (left) and then quantified (right). There was a does-dependent increase in expression of Cyr61 protein. Cyr61 expression was normalized by GAPDH expression. Results shown are the mean ± SD of three independent experiments. * P<0.05; ** P<0.01.

The mRNA expression and protein production of Cyr61 in Müller cells treated with different concentration of glucose were evaluated by RT-qPCR and Western blot respectively. Expression of Cyr61 mRNA in Müller cells treated with 10 mM glucose was observed for 0–24 h, and we found a time-dependent increase in Cyr61 mRNA expression (Figure 2A). There was a sharp increase after 8 h (*P* = 0.004) and Cyr61 mRNA expression after 24 h was about 4-fold higher than that of 0 h (*P* = 0.002). Cyr61 mRNA level in Müller cells exposed to different glucose concentrations (5–20 mM) for 8 h was examined. The Cyr61 mRNAl was increased in a dose-dependent pattern. The Cyr61 mRNA expression at 10 mM glucose was 4.7-fold higher than the control group (*P* = 0.006). The Cyr61 mRNA expression was largely increased (up to 6-fold) in cells exposed to 20 mM glucose, which was significantly higher than the control group (*P* = 0.003) (Figure 2B). Cyr61 protein production also showed a dose-dependent increase. When glucose concentration was 10 mM, the Cyr61 expression was higher than the control group. The difference was significant (*P* = 0.02). The Cyr61 expression of Müller Cells exposed to 20 mM was 9-fold higher than the control group (*P* = 0.003) (Figure 2C).

### Effect of Cyr61 on the viability, migration and apoptosis of Müller cells

**Figure 3:**
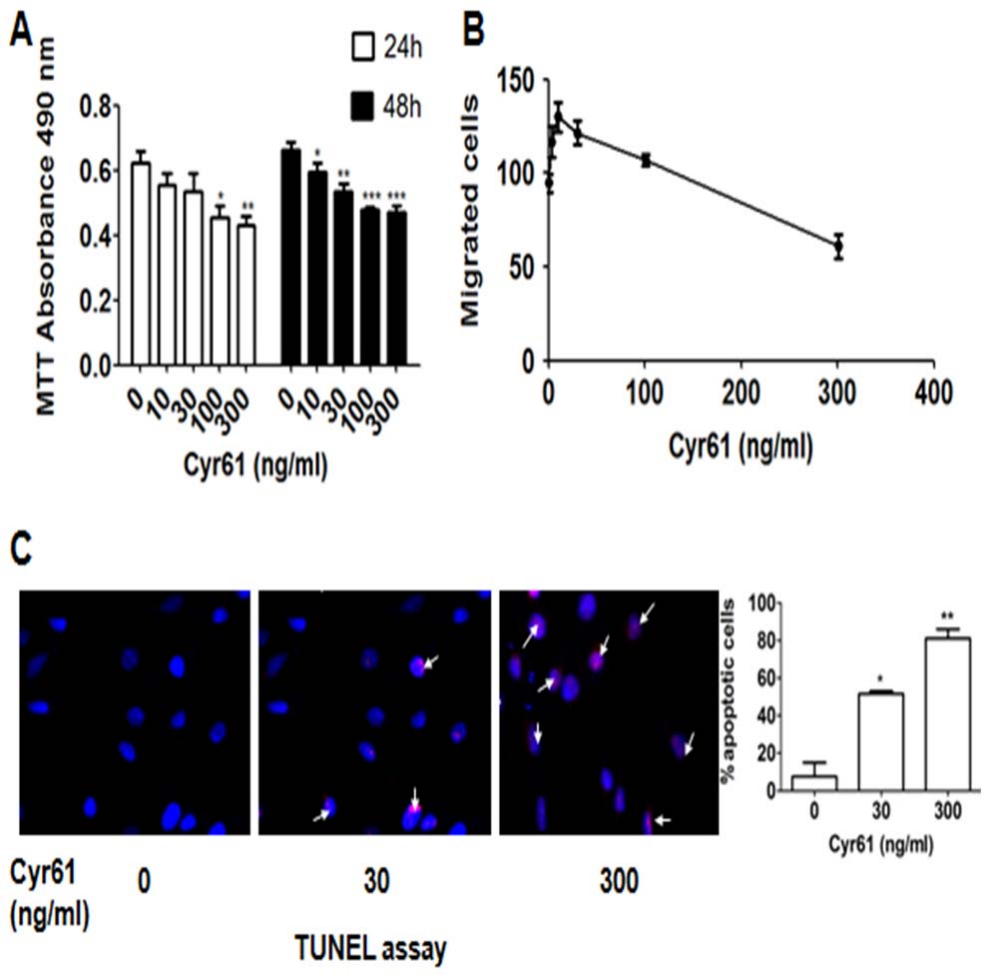
Effect of Cyr61 on the viability, migration and apoptosis of Müller cells was observed. A) Müller cells viability was determined with MTT assay after 24 h and 48 h. Treatment of Cyr61 decreased Müller cells viability. B) Müller cell migration in response to Cyr61 treatment was measured using Transwell assay. Note that cell migration was decreased under high concentration of Cyr61. C) Müller cells apoptosis in response to Cyr61 treatment (0, 30, 300 ng/ml) was measured by TUNEL assay. Apoptosis of Müller cells was promoted by high level of Cyr61. Results shown are the mean ± SD of three independent experiments. * P<0.05; * P<0.01; *** P<0.001.

Different Cyr61 concentrations (0–300 ng/ml) were used to treat Müller cells for 24 h and 48 h. Müller Cell viability decreased with the increase of Cyr61 concentration (Figure 3A), and the difference was significant (one-way ANOVA, F = 3.690, *P* = 0.028; F = 15.007, *P* = 0.000). When Müller cells were incubated with various concentrations of Cyr61 for 24 h, cell viability was significantly decreased at 100 ng/ml and 300 ng/ml. When Müller cells were incubated with various concentrations of Cyr61 for 48 h, cell viability started to decrease at 10 ng/ml. When Cyr61 reached to 30 ng/ml, cell viability was significantly decreased. The effect of Cyr61 on Müller cells migration was determined using Transwell assay. The values were assessed by the mean number of migrated cells. Müller cells' migration was significantly decreased when Cyr61 was 300 ng/ml (Figure 3B), the difference was significantly compared with the control group (one-way ANOVA, F = 122.402, *P = *0.000). TUNEL assay was applied to examine the apoptosis of Müller cells exposed to Cyr61. When Cyr61 concentration was 30 ng/ml, Cyr61 started to promote Müller cells apoptosis. At the concentration of 300 ng/ml, Cyr61-induced Müller cells apoptosis was obvious (one-way ANOVA, F = 49.153, *P = *0.005) (Figure 3C). We also did the correlation analysis among cell viability, apoptosis and migration by Pearson Correlation, and found that the cell migration was related to the cell viability and apoptosis, with correlation coefficient r = 0.358 and r = −0.997. The correlation coefficient r between cell viability and apoptosis was −0.978.

## Discussion

In this study, it was revealed that high glucose significantly elevated Cyr61 expression in Müller cells with dose-dependent and time-dependent manner. It was also found that high level of Cyr61 contributes to the degeneration of Müller cells by suppressing the viability, inhibiting the migration and promoting the apoptosis of the retinal Müller glial. According to the literature available, there are little published papers showing the mutual effect between that high expression of Cyr61 and Müller cells in DR. In addition, we found that Cyr61 levels in vitreous fluid in patients with PDR were significantly higher than those without DR.

### Increased levels of Cyr61 in vitreous fluid are associated with the patients with DR

We found that vitreous levels of Cyr61were elevated in patients with PDR when compared with non-diabetic patients, which was similar with other researches [19], [24]. Although the patient numbers in each group in this vivo study were not large, but they were sufficient for the statistical significant analysis. As an angiogenesis factor, Cyr61 was usually thought to play a negative part in pathogenesis of DR, like promoting the neovascualization. However, in a mouse model of oxygen-induced retinopathy, Hasan and his team found that expression of Cyr61 in the vitreous humor has significant beneficial effects in repairing damaged retinal vasculature [25]. Deriving from our study, we proposed another potential advantage of high level of Cyr61 in suppressing the retinal gliosis in DR, which will be discussed in detail later.

### Cyr61 is expressed by human Müller cells

Cyr61 is reported to be secreted mainly by retinal endothelial cells and pericytes [22]. However, there are few studies published demonstrating that the expression of Cyr61 is up-regulated in retinal Müller cells in DR, and the underlying mechanism still remains unclear.

The role of Cyr61 is complex in retinal pathological progress. On one hand, Cyr61 has potent angiogenic activity upon endothelial cells and induces neovascularization [19], [24], [26]. Study by You [20] stated that Cyr61 induced expression of VEGF and vice versa, and anti-Cyr61 or anti-VEGF could inhibit the effects of both Cyr61 and VEGF. On the other hand, as an endogenous pro-survival factor for photoreceptors, elevated Cyr61 could contribute to the complex repertoire of neuroprotective activities generated by RMG and RPE cells [27].

### Effect of Cyr61 on the degeneration of Müller cells

We also showed that increased Cyr61 inhibited viability and promoted apoptosis of Müller cells. However, pathological sequence of Müller cell apoptosis remain unclear [28]. The study by Hammes [29] indicated diabetes induces the programmed cell death in retinal Müller cells of early diabetic rats. He argued that it is the transdifferentiation of Müller cells into the phenotype with low affinity of nerve growth factor receptor that accounts for the apoptosis [30]–[33].

Studies have shown that Cyr61 promotes endothelial cell proliferation and neovascularization. We are interested in the mechanism of the different effects of Cyr61 on endothelial cells and Müller cell. We hypothesized that it might be a seemingly protective reaction of the retina. Under the high glucose, the retina increases the expression of the Cyr61 to help to improve the perfusion of the retina by promoting endothelial cells, while at the same time to prevent the retinal fibrosis by inhibiting the reactive Muller cells.

The inhibitory effect of Cyr61 on the viability of Müller cells is seemingly contributing to both damage and protection of the retina. On one aspect, retinal Müller cells could help to maintain the homeostasis of the retinal microenvironment, such as reducing the concentration of glutamate, potassium and thus liquid in retina [28], [34]–[36]. And Müller cells could also secret the neurotrophic factors to prevent the neurons from degeneration [12], [37], [38]. So the apoptosis of the Müller cells would lead to the increasing vulnerability of the retinal neurons. On the other aspect, the hyperreaction of Müller cells partially contributes to the retinal scar, like PDR, which drastically damages the normal structure and functions of the retina. Based on our study, Cyr61 could suppress the viability of Müller cells, and thus could be beneficial in preventing the retina from glial proliferation. A similar phenomenon presents in skin wounds is that Cyr61 accumulates in the granulation tissue as myofibroblasts proliferate, and eventually reaches a sufficiently high level to drive the myofibroblasts into senescence, reducing the risk of fibrosis during wound healing [13]. It is also revealed in our study that the Cyr61 promotes the migration of the Müller cells at low concentration, but inhibits it at high concentration. When there is a retinal injury, the Müller cells would migrate to the site and help to repair [39]–[41]. At the same time, Cyr61 is highly expressed at sites of inflammation and wound repair as well, synthesize extracellular matrix to maintain tissue integrity and to promote regeneration of parenchymal cells [42]. So there might be a kind of synergic effect of the Müller cells and Cyr61 in retinal wound healing. However, excessive migration of Müller cells into vitreous body, subretinal layer, and other spaces originally occupied by neurons might lead to catastrophe to the retina, such as tractional retinal detachment. According to our study, Cyr61 at high level could suppress the migration and proliferation of the Müller cells, protecting the retina from scaring.

It was found that the cell migration was related to the cell viability and apoptosis, with correlation coefficient r = 0.358 and r = −0.997. As a result, we couldn't rule out the effect of low viability or high apoptosis on the reduction of the cellular migration. In another words, high Cyr61 might indirectly inhibit the Müller cells's migration by decreasing the cellular viability or promoting the cellular apoptosis.

Further investigations are necessary to identify the role of the interaction between Cyr61 and Müller cells in vivo using diabetic animal models. Besides, it is interesting to explore the potential signaling pathways of Müller cells apoptosis by Cyr61. And also, experimental evidence should be obtained to verify the potential patho-physiological pathway mentioned above.
